# What's in a band? The function of the color and banding pattern of the Banded Swallowtail

**DOI:** 10.1002/ece3.6034

**Published:** 2020-01-28

**Authors:** Eunice J. Tan, Bodo D. Wilts, Brent T. K. Tan, Antónia Monteiro

**Affiliations:** ^1^ Yale‐NUS College Singapore City Singapore; ^2^ Adolphe Merkle Institute University of Fribourg Fribourg Switzerland; ^3^ Department of Biological Sciences National University of Singapore Singapore City Singapore

**Keywords:** coincident disruptive coloration, differential blending, disruptive coloration, *Papilio*, predation, signaling

## Abstract

Butterflies have evolved a diversity of color patterns, but the ecological functions for most of these patterns are still poorly understood. The Banded Swallowtail butterfly, *Papilio demolion demolion*, is a mostly black butterfly with a greenish‐blue band that traverses the wings. The function of this wing pattern remains unknown. Here, we examined the morphology of black and green‐blue colored scales, and how the color and banding pattern affects predation risk in the wild. The protective benefits of the transversal band and of its green‐blue color were tested via the use of paper model replicas of the Banded Swallowtail with variations in band shape and band color in a full factorial design. A variant model where the continuous transversal green‐blue band was shifted and made discontinuous tested the protective benefit of the transversal band, while grayscale variants of the wildtype and distorted band models assessed the protective benefit of the green‐blue color. Paper models of the variants and the wildtype were placed simultaneously in the field with live baits. Wildtype models were the least preyed upon compared with all other variants, while gray models with distorted bands suffered the greatest predation. The color and the continuous band of the Banded Swallowtail hence confer antipredator qualities. We propose that the shape of the band hinders detection of the butterfly's true shape through coincident disruptive coloration; while the green color of the band prevents detection of the butterfly from its background via differential blending. Differential blending is aided by the green‐blue color being due to pigments rather than via structural coloration. Both green and black scales have identical structures, and the scales follow the Bauplan of pigmented scales documented in other *Papilio *butterflies.

## Graphical abstract

Our research aims to address fundamental questions about the ecology and evolution of colour patterns by using the Banded Swallowtail butterfly, a mostly black butterfly with a greenish‐blue band that traverses the animal from one wing apex to the other. We performed field experiments with paper model replicas with variations in band shape and band colour, to understand the protective benefits of the transversal band and of its green‐blue colour. We found that wildtype models were the least preyed upon compared with all other variants, and propose that the shape of the band hinders detection of the butterfly's true shape through coincident disruptive coloration, while the green colour of the band prevents detection of the butterfly from its background via differential blending.

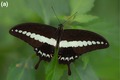

## 1 INTRODUCTION

Animals have evolved a bewildering diversity of color patterns. Some of these color patterns are used to signal to the opposite sex (e.g., Baldwin & Johnsen, 2009; Engelking, Roemer, & Beisenherz, 2010; Lim, Land, & Li, 2007), but perhaps the majority help in providing protection from potential predators (e.g., see reviews by Stevens and Merilaita (2011), Merilaita, Scott‐Samuel, and Cuthill (2017) and Cuthill (2019)). The visual camouflage strategies employed by animals to escape detection by predators are diverse, and it can be challenging to identify how these signals serve their protective function.

Multiple mechanisms involved in animal camouflage have been further dissected in the last decade. A key form of concealment is crypsis, which comprises of traits that prevent the initial detection of the animal (Stevens & Merilaita, 2009a, 2011). Disruptive coloration is a strategy of crypsis, which makes an animal difficult to detect and/or recognize by predators by disrupting recognizable features of the animal (Cott, 1940; Cuthill et al., 2005; Endler, 2006; Stevens & Merilaita, 2009b; Troscianko, Skelhorn, & Stevens, 2017; Webster, Hassall, Herdman, Godin, & Sherratt, 2013). Here, we follow the definitions of Stevens and Merilaita (2009b) to define two specific subprinciples of disruptive coloration—*differential blending* and *coincident disruptive coloration*. As natural backgrounds can be variable, *differential blending* allows at least some of the colors of a pattern to blend into the background, thus disrupting the animal's shape (Cott, 1940; Espinosa & Cuthill, 2014; Stevens & Merilaita, 2009b). In *coincident disruptive coloration*, continuous patterns such as bands could cover different but adjacent body parts of an animal, thus masking otherwise potentially revealing body parts of the animal (Cuthill & Székely, 2009; Stevens & Merilaita, 2009b).

In order to understand how a particular signal confers protection to a prey species, it is useful to work with prey species that display prominent signals such as butterflies. Swallowtail butterflies (Lepidoptera: Papilionidae), in particular, are large and colorful species distributed worldwide that display a large diversity of wing color patterns (Aubert, Legal, Descimon, & Michel, 1999). While numerous studies have examined visual signals displayed by *Papilio* larvae (Prudic, Oliver, & Sperling, 2007; Tullberg, Merilaita, & Wiklund, 2005), little is known about the defensive strategies of *Papilio* adults beyond studies that have examined convergent wing pattern elements used in both Batesian and Müllerian mimicry rings (e.g., (Kitamura & Imafuku, 2015; Ohsaki, 1995; Palmer et al., 2018; Uésugi, 1996)).

**Figure 1:**
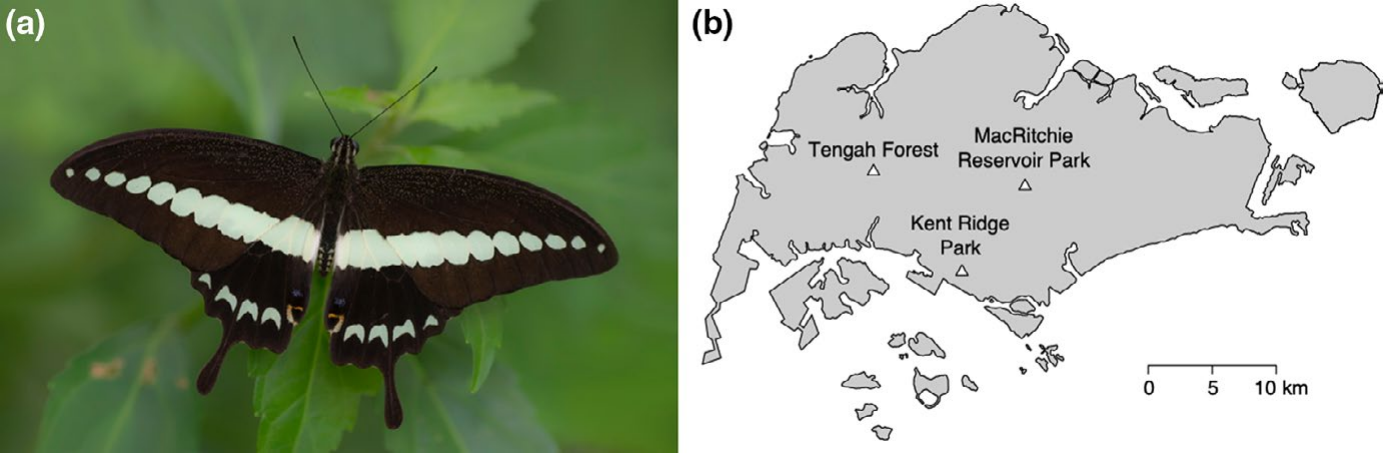
(a) Image of the Banded Swallowtail, *Papillio demolion* in the wild in Singapore and (b) the locations of our experimental sites in Singapore. White triangles represent the locations of the field sites, with the scale of the map on the bottom right. *Papilio demolion* image credit: Sin Khoon Khew

Here, we investigate the function of the color patterns of the Banded Swallowtail, *Papilio demolion demolion*, to try and tease apart the effects of two subprinciples of disruptive coloration—*differential blending* and *coincident disruptive coloration* on this butterfly. We examine whether either of these two disruptive coloration strategies is being used by this species. The Banded swallowtail is mostly black butterfly with a dorsal conspicuous greenish‐blue transversal band that extends from the apex of the forewing to the inner margin of the hindwing, on one site, and continues, on the other side of the body, to the apex of that forewing, creating an uninterrupted band of color across the animal (Figure 1). There is also a series of similarly colored chevrons along the hindwing margins (Figure 1). Both sexes look alike. This species lives across South East Asia and Australia, and while it can be found in forest edges and clearings, it is most commonly observed in primary and secondary forests and nature reserves (Khew, 2015; Kirton, 2014). This species is an active and fast flier, observed flying in the forest understorey and in the open, feeding on flowers of shrubs and trees in mid to late morning (Khew, 2015; observed by ET and AM at one of our field sites). When viewed in the dim light of a forest the blue‐green color of the transverse band could be enhancing the contrast of this banding pattern with the black of the background color (Endler, 1993), and this would help break up the shape of the butterfly (Troscianko et al., 2017). Alternatively, the blue‐green color could help reduce predator detection via background matching, as the green color of the band could match the color, lightness, and pattern of the surrounding green vegetation in the background. We hypothesized that the transverse band of the Banded Swallowtail may be a form of coincident disruptive coloration that disguises the shape of the butterfly, preventing recognition by predators, while its greenish‐blue color functions to disrupt the butterfly shape through differential blending.

To test the protective benefit of both the transversal band as well as its blue‐green color, we constructed four different paper model variants of the Banded Swallowtail and tested how frequently each model got attacked by predators in the native habitat of the butterflies. Artificial paper models have been used in other studies involving predation and mate preferences to great effect, showing that these models are effective imitations of their real counterparts (Finkbeiner, Briscoe, & Reed, 2012; Ho, Schachat, Piel, & Monteiro, 2016; Palmer et al., 2018; Stevens, Hardman, & Stubbins, 2008; Wee & Monteiro, 2017). To test the protective benefit of the transversal band, we created a variant model where the continuous band was shifted to highlight the shape of the forewings and hindwings, rather than transverse them. To test the protective benefit of the green‐blue color, we created a grayscale variant of the wildtype. To test the protective benefits of both the transversal band and its green‐blue color simultaneously, we created a grayscale variant with a distorted transversal band. These four different types of paper models were placed in the field with mealworm baits. If the transversal band of the wildtype is a form of coincident disruptive coloration, models with distorted bands should suffer greater predation than models with the transverse band. If the blue‐green color of the wildtype serves as a form of differential blending, grey banded models should suffer greater predation than blue‐green banded models with the same luminance. Finally, if the color pattern of the wildtype serves as both a form of coincident disruptive coloration and a form of differential blending, grey‐distorted models should fare worse than all other models.

Because the origin of the color‐producing mechanisms of the green‐blue color in this species of butterfly is so far unknown, we also examined the ultrastructure of the blue‐green scales with a scanning electron microscope and compared their pigmentation relative to the flanking black scales in the same wing using absorbance measurements. We hypothesized that if the butterfly coloration has evolved to background match its environment to reduce detection and thus predation, the blue‐green color of the wing scales should originate from pigmentary absorption rather than to structural colors. This is because structural colors are usually iridescent, that is, the color changes depending on the observation and illumination angle. Pigmentary colors retain the same color regardless of where a predator might be located (Kinoshita, Yoshioka, & Miyazaki, 2008; Srinivasarao, 1999). Blue and green colors in butterfly wing scales are usually produced by interference of light at photonic structures formed by fine, repeated, cuticular structures on the scale, rather than via pigmentation (Kinoshita et al., 2008; Srinivasarao, 1999). In this case, however, we show that pigments are involved in producing this blue‐green color resulting in the butterfly having a matt appearance with no iridescence.

## 2 MATERIALS AND METHODS

### 2.1 Reference specimens

Preserved specimens of the Banded Swallowtail butterfly were obtained via Ebay from an insect collector, Andreas Muller, from Austria.

### 2.2 Preparation of paper butterfly models

Our paper models imitated the Banded Swallowtail at its natural resting position, displaying its dorsal wing patterns (Figure 3). A half image of the Banded Swallowtail was edited in Photoshop CC 2014 to create a wildtype and three color pattern variants on a single side of the butterfly. This side of the butterfly was then mirrored so that the left and right wings would be identical to each other. Models were printed on a HP Deskjet 2540 printer with HP61 ink, on HP printer paper, to the scale of the actual butterflies (Khew, 2015), with a wingspan of 75 mm. The paper models were then soaked and covered with paraffin wax to render them resistant toward bad weather conditions (Wee & Monteiro, 2017). Larvae of the beetle *Tenebrio molitor* (mealworms) were attached as baits to the paper models. As live baits, mealworms are more effective compared with other choices such as pastry, clay or plasticine, because they draw a higher number of attacks in a short amount of time (Ho et al., 2016). Mealworms were placed on the middle, underside area of each model, firmly stuck between two pieces of Blu‐Tack. The mealworm was partially visible from the top view of the model, as the mealworm protruded from the posterior end of the butterfly. To prevent the mealworm larva from being attacked by ants and other crawling insects which are not natural predators of the Banded Swallowtail, the model was elevated with a piece of Blu‐Tack placed on the underside of the left wing. Insecticide (Baygon Multi‐Insect Killer) was applied to the Blu‐Tack beforehand. Although the insecticide had a smell, as all models were similarly treated, we do not expect olfactory cues from the applied insecticide to affect the predation on models differently.

### 2.3 Model color and scale color reflectance and absorbance measurements

Butterfly wings and complete paper models were imaged under a Zeiss Axioscope A1 light microscope (Zeiss) with reflected and transmitted light using a Point Grey Grasshopper 3 camera (FLIR). Reflectance spectra were measured by placing one end of a fiber optic cable in the far‐field of the detection pathway in a position confocal to the front focal plane of the objective, which guided the light to an Ocean Optics QE Pro spectrometer (Ocean Optics). A white diffuser (Ocean Optics) served as a standard. To test whether pigments were present in both green and black scales, scales were removed from the wing, immersed in refractive index oil (Cargille Labs, *n* = 1.55) and light transmittance through the scale was measured (i.e., we measured the absorbance of the two types of colored scales).

### 2.4 Field sites and experimental setup

Field experiments were performed at three secondary forest sites in Singapore (Figure 1—a) Kent Ridge Park (01°17′N, 103°46′E), (b) Tengah forest (01°21′N, 103°43′E), and (c) MacRitchie Reservoir Park (01°20 N′, 103°49′E)—during December 2016 to February 2017. Banded Swallowtail butterflies have been observed at site B by two of our authors on several occasions (AM and ET) and have been observed at several secondary forest locations in Singapore by citizen scientists (iNaturalist). Known host plants of the Banded swallowtail (Khew, 2015) were observed at all three sites—*Luvunga crassifolia* and *Melicope lunu‐ankenda*. Avian insectivores such as babblers (Timaliidae), bulbuls (Pycnonotidae), cuckoos (Cuculidae), drongos (Dicruridae), flowerpeckers (Dicaeidae), and kingfishers (Alcedinidae) are known in secondary forests in Singapore (Jeyarajasingam, 2012; Ng, Corlett, & Tan, 2011), and a few of these avian insectivores were observed at site B on several occasions, by one of our authors (ET).

At each site, all four model types were placed simultaneously and in identical numbers to compare predation rates across all models under the same condition. A total of 660 models of the four types were placed in the field. The number of models placed varied across sites—25 models of each type at site A, 50 of each type at site B, and 90 models of each type at site C. Models were placed in sets consisting of one model type per set, with sets placed 1–2 m apart. Individual models in each set were placed on the leaves of shrubs, at least 0.5 m apart from one another. As butterflies frequently aggregate at flowers or salt pools (puddling; documented in various sources, for example, Arms, Feeny, & Lederhouse, 1974; Matter & Roland, 2002; Molleman, 2010), it is not uncommon to have higher butterfly densities in certain areas. We do not expect potential avian predators to particularly favor one model over another just because of the proximity, as model types were equally represented in each set. Models were placed in the field on Day 1, left for 4 days, and predation was scored daily from Day 2. A model was considered to have been preyed on if the mealworm attached to it was partially or fully consumed. Attacked models were not replaced or removed until the end of the experiment, as in Ho et al. (2016) and Wee and Monteiro (2017).

### 2.5 Statistical analysis

To test for differences in predation of the models over time, we performed survival analysis of the models by fitting a cox model containing mixed effects. We examined the survival of variants (Wt‐distorted, Grey and Grey‐distorted) against Wt over time, with predation as the response variable, and site as a random effect variable, using the *coxme* package (Therneau, 2015) in *R* v. 3.6.0 (R Core Team, 2019). To visualize the survival probability of the various butterfly models over time, we plotted survival curves of the various models using the *survminer* package (Kassambara & Kosinski, 2019) in *R*. Next, we plotted cumulative incidence curves using the *survminer* package (Kassambara & Kosinski, 2019) in *R* to visualize the relevant confidence intervals of the individual butterfly models over time. To estimate the relative contribution of band color, band shape, and the interaction between these factors on predation risk, we fit a generalized linear mixed‐effects model (GLMM) to the data using *lme4* package (Bates, Maechler, Bolker, & Walker, 2015) in *R*. We used a binomial error distribution, with prey status (attacked or not) on the final day of the experiment (day 4) as the response variable, with band color and band shape as interacting fixed effects, and site as a random effect. As there was no interaction effect of band color and shape, we repeated the GLMM analysis without interaction effects, to simplify the model. We reported the odds of predation, derived from exponentiating the coefficients obtained from the models.

## 3 RESULTS

### 3.1 Banded swallowtail wing and scale color measurements

**Figure 2:**
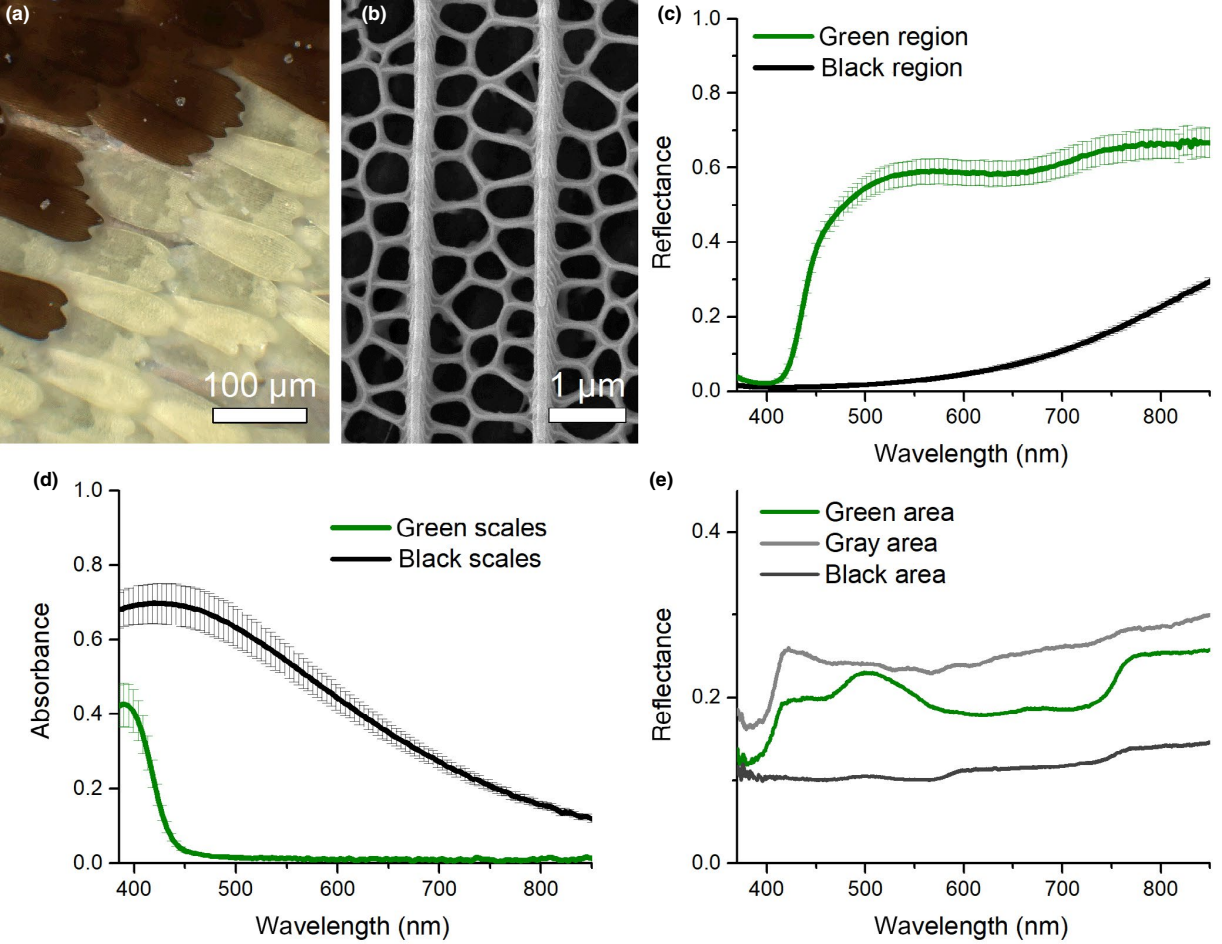
Optics of the butterfly and paper models. (a) Scale lattice, (b) representative SEM image of green and black scales, (c) reflectance of different colored wing patches, (d) single scale absorbance spectra of *Papillio demolion*, and (e) reflectance spectra of butterfly models

The Banded Swallowtail has black and green regions where single colored scales imbricate the wing like shingles on a roof (Figure 2a). Reflectance spectra of black and green scales show that the black scales are low in reflectance, throughout the whole visible wavelength range, suggesting the presence of melanin (Figure 2c), the absorbance spectra of single scales immersed in refractive index fluid supports this hypothesis (Figure 2d). On the contrary, the green scales are rather broadband reflectors. Their reflectance spectra feature a minimum in the UV region, a strong rise in reflectance at ~430 nm, and a second rise at wavelengths above 650 nm. This suggests the presence of (at least) one UV‐absorbing pigment in these scales. Absorbance measurements confirm the presence of an absorbing pigment with a peak absorbance at approximately 395 nm (Figure 2d). The reflectance does not show a narrow reflectance band typical for photonic nanostructures (Srinivasarao, 1999; Trzeciak, Wilts, Stavenga, & Vukusic, 2012), suggesting a sole pigmentary origin of the coloration. To confirm this, we performed SEM of the green and black scales. Indeed, the structure of both, green‐ and black‐colored, scales is identical and the scales follow the *Bauplan* of pigmented scales in *Papilio* butterflies (Ghiradella, 1985, 2010) (Figure 2b), as also seen in the pigmentary scales of closely related *Parides* butterflies (Wilts, Ijbema, & Stavenga, 2014).

The reflectance of the butterfly models (Figure 2e) used in the predation experiments approaches the chromatic contrast of the butterfly sample: The green‐colored wildtype model's bands are higher in reflectance with a broad reflectance band between 480 and 550 nm, giving these models a cyan‐green color that closely matches the natural sample in its hue. The precise reflectance shape is different from the butterfly and shows a rather pronounced reflectance band in the blue‐green wavelength range rather than a broader reflectance peak that is levelling off. The Grey model's band is also functioning as a broadband reflector but it misses the characteristic rise in reflectance of the wildtype green band, allowing Grey and wildtype models to differ primarily in hue rather than in color intensity. The black background in all models is more reflective than their natural counterpart, likely due to the wax layer adding an extra smooth dielectric layer that increases light reflectance. The hue and spectral shape of the black color in the models are, however, low, thus maintaining a contrast that is very similar to the biological sample throughout the visible wavelength range.

### 3.2 Predation on models

**Figure 3:**
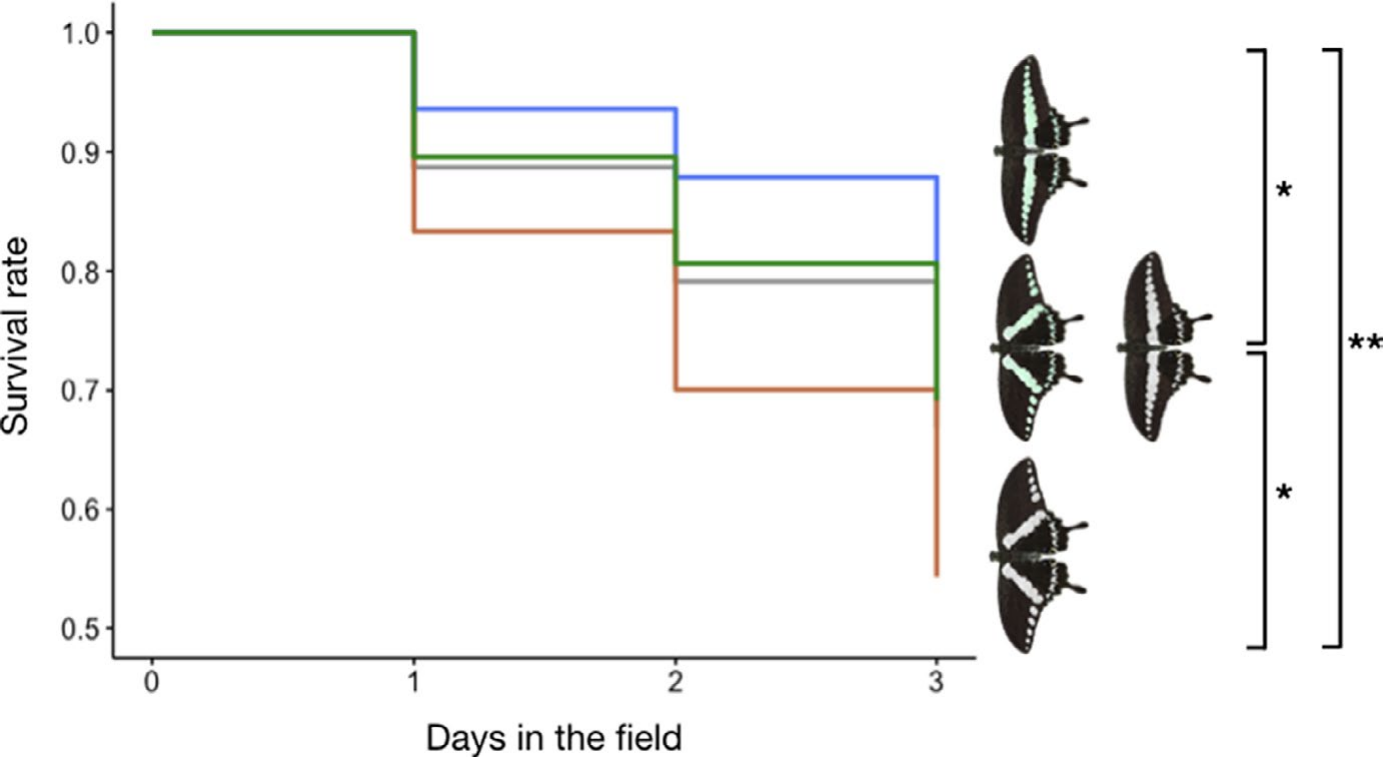
Survival curves of the various models in the field. Blue, green, gray, and orange lines represent the survival rates of the wildtype, Wt‐distorted, Grey, and Grey‐distorted models respectively. Images to the right of the curves illustrate the various models used. Wildtype models fared significantly better than Wt‐distorted, Grey, and Grey‐distorted models. Grey‐distorted models also had significantly lower survival compared with Wt‐distorted and Grey models. Wt‐distorted and Grey models did not differ significantly from each other. *P*‐values are indicated by asterisks: *p < .05; **p < .001

**Figure 4:**
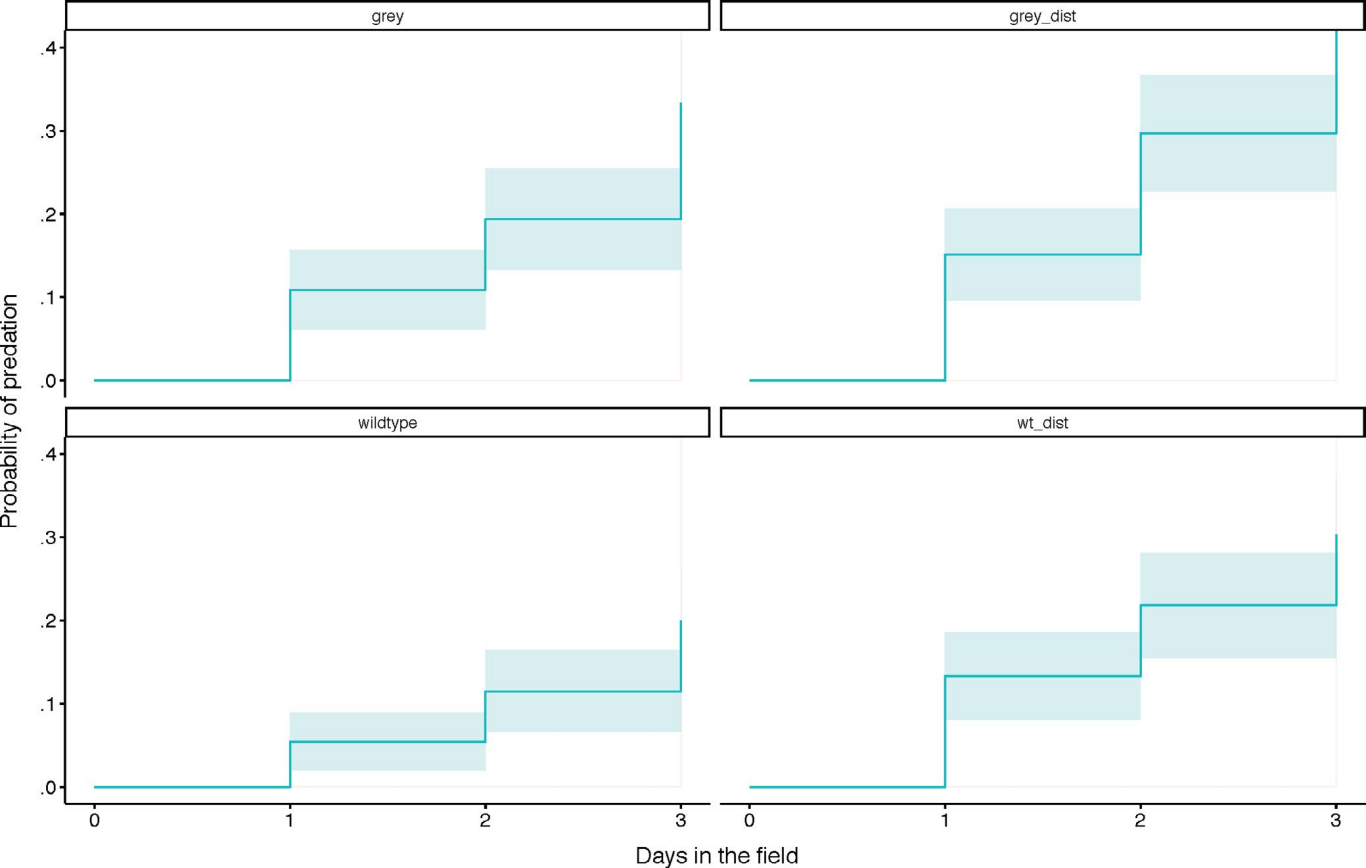
Cumulative incidence curves depicting the predation probability of the various model types. The solid line indicates the probability of predation on a specific model type over time, and the colored shading represents the corresponding confidence interval

Our results indicated that the three variants (Wt‐distorted, Grey, Grey‐distorted) suffered higher predation than the wildtype over the course of the experiment (Figure 3). Figure 3 clearly shows that the relative survival across models was consistent over time, with the wildtype consistently faring the best, followed by Grey and Wt‐distorted models, then Grey‐distorted models. Compared with the wildtype, Wt‐distorted models were 1.68 times more likely to be preyed on, followed by Grey models, which were 1.86 times more likely to be preyed, with Grey‐distorted suffered the highest predation at 2.86 times (Table 1). Grey and Wt‐distorted models were significantly less preyed on compared with Grey‐distorted models, at 0.65 and 0.58 times respectively (Table 1). Figure 4 further illustrates the probability of predation with the corresponding confidence intervals for the individual model types. The results of the simplified GLMM indicate that band distortion and grayscaling of the band negatively affected the predation of model types in almost equal measure. Distorted bands (*z* = 3.25, *p* = .001) were 1.77 times more likely to cause predation, while grayscaled bands (*z* = 4.08, *p* < .001) were 2.05 times more likely to cause predation. These two factors did not interact.

**Table 1:** Results of survival analysis by fitting a cox model containing mixed effects

Pairwise comparison with	Models	Hazards ratio (95% confidence interval)	*z*‐value	*p* value
Wildtype	Wt‐distorted	1.68 (1.08–2.60)	2.31	2.1 × 10^–2^
	Grey	1.86 (1.21–2.87)	2.82	4.7 × 10^–3^
	Grey‐distorted	2.86 (1.90–4.30)	5.03	4.9 × 10^–7^
Grey‐distorted	Wt‐distorted	0.58 (0.41–0.83)	−2.92	0.0035
	Grey	0.65 (0.46–0.92)	−2.42	0.016

## 4 DISCUSSION

Our results clearly show that the transversal greenish‐blue band of the wildtype is more effective in deterring predation than a nontransversal band or a gray colored band of either type. The effectiveness of the two elements—the transversal band and the green‐blue color—are relatively similar, with both the Wt‐distorted and Grey models having similar, higher risks of predation (*HR*: 1.68 and 1.86 respectively) compared with the wildtype (Figure 3; Table 1). The Grey‐distorted models suffered the greatest predation compared with the wildtype, suggesting that the double loss of the transversal band, and the green‐blue color negatively affected the survival of the models.

The distortion of the transverse band is clearly affecting predation risk. We suggest that this indicates that the transverse band confers a protective advantage in the form of coincident disruptive coloration, as it disrupts the true shape of the butterfly. The presence of bands along the wing margin in another butterfly species, *Anartia fatima*, while of a different color and position on the wing compared with the Banded Swallowtail, also serves to reduce predation, through creating a false boundary (Seymoure & Aiello, 2015). Variants where the band was shifted to form an outline of the wings of *A. fatima*, or as a discontinuous edge along the wing boundary, both resulted in lower survivorship compared with wildtype models. In yet another invertebrate system, the yellow‐colored bands of an orb‐weaving spider function to obscure the outline of the spider to visually hunting spiders (Hoese, Law, Rao, & Herberstein, 2006). Bands on yet another taxa, fish, have shown to be associated with their speed of movement, body types, and habitat (Barlow, 1972). Longitudinal bands occur more frequently on the eye lines of faster moving, slender species associated with bottom living, while vertical bands occurred more frequently on sharply turning, deep‐bodied fishes that live close to their substrate (Barlow, 1972). The occurrence of longitudinal bands could be an effective form of coincident disruptive coloration for fast‐moving species across a range of taxa, as both the longitudinally striped fishes and the Banded Swallowtail are fast‐moving animals.

The origin of the different colors found on the wings of the Banded Swallowtail butterflies relies solely on pigmentation. While many colors in *Papilio* butterflies are structural, with nanostructures as diverse as ridge multilayers and photonic crystals in the scale lumen (Huxley, 1975; Ingram & Parker, 2008; Kolle et al., 2010; Wilts et al., 2014), the Banded Swallowtail butterfly employs a mix of pigments to create the greenish appearance of the scales on the wing.

The importance of the green‐blue color of the band is also evident, with the higher predation on the Grey models compared with the wildtype models. We propose that this color functions in differential blending, but our experiment cannot dismiss alternative possibilities (discussed below). The blue‐green color may help the butterfly better blend in with the surrounding vegetation and/or enhance the contrast of the pattern, and better breakup the outline (Troscianko et al., 2017), as blue‐green colors in a signal increases contrast against the background when viewed in a forest shade (Endler, 1993). The green‐blue band of the Banded Swallowtail also acts as a UV‐absorber (Figure 2) and this produces a striking contrast against the black wings, which reflect some UV (not shown).

We also considered whether perhaps the green‐blue color could be functioning as a warning color. Similar to the Banded swallowtail, other species of butterflies such as *Parides* sp. have green patterns, often in combination with black wings, that are believed to function as aposematic signals (Pinheiro, 2008). Dissimilar to other swallowtail butterflies, however (Euw, Reichstein, & Rothschild, 1968; Wilmoth & Fordyce, 2019), the Banded Swallowtail is unlikely to sequester toxins from its host plants, as the larvae feed on the leaves of nontoxic plants—*L. crassifolia*, *Luvunga scandens*, *Acronychia peduculata*, *Melicope luna‐ankenda,* and *Citrus* spp. (Corbet & Pendlebury, 1956; Ek‐Amnuay, 2012; Khew, 2015). However, not all warning colors signal unpalatability, some of these colors could be used to signal unprofitability. Pinheiro, Freitas, Campos, DeVries, and Penz (2016) showed that warning coloration in butterflies can function as a signal to indicate difficulty of capture by insectivorous birds. As the Banded Swallowtail is a strong flier, its blue‐green band may serve as a warning color to signal unprofitability to insectivorous birds.

Both the color and the band of this butterfly may help it form a mimicry ring with other local species that share similar traits (Joshi, Prakash, & Kunte, 2017; Mallet & Gilbert, 1995). Other species of palatable *Papilio* butterflies are known Batesian mimics of unpalatable, aposematic butterflies (Chai, 1986; Kunte, 2009). The Common Bluebottle, *Graphium sarpedon luctatius*, is a common species found in forests and forest edges (Khew, 2015; Kirton, 2014), and may be involved in a mimicry ring with the Banded Swallowtail. Both butterflies have green‐blue bands across their otherwise black dorsal surfaces. Like in the Banded Swallowtail butterfly, a pigment mix results in the green‐blue color in *G. sarpedon* (Stavenga, Giraldo, & Leertouwer, 2010). The Common Bluebottle has a green‐blue macular band which runs from the apex of the forewing to the inner margin of the dorsal hindwing of the butterfly. Two of our authors (ET, AM) have observed the Common Bluebottle and the Banded Swallowtail butterflies at site C, but the chemical defences of both these butterfly species are currently unknown. Our predation experiments cannot reject an alternative hypothesis that wildtype models were least attacked due to aposematism or mimicry, instead of crypsis due to disruptive coloration. Future experiments could test the function of crypsis against aposematism by placing variant models in both natural cryptic background and a standardized artificial gray background, following previous studies (Barnett, Michalis, Scott‐Samuel, & Cuthill, 2018; Wüster et al., 2004).

Together, the presence of the transversal band and the green‐blue color resulted in the lowest predation risk across our models. We suggest that the transversal band and the green‐blue color positively affected the survival of the models through differential blending and perhaps a combination of coincident disruptive coloration as well as warning coloration that signals unprofitability.

## CONFLICT OF INTEREST

None declared.

## AUTHOR CONTRIBUTIONS

AM conceived the project; BT and ET performed the fieldwork; BDW performed the ultraspectral and spectroscopic analyses of the reference and model butterfly colors; ET performed the statistical analyses; ET, BDW, and AM wrote the manuscript.

## Data Availability

Data from this manuscript are deposited in Dryad, DOI (https://doi.org/10.5061/dryad.qz612jm9p).
